# RSS Indoor Localization Based on a Single Access Point

**DOI:** 10.3390/s19173711

**Published:** 2019-08-27

**Authors:** Akis Kokkinis, Loizos Kanaris, Antonio Liotta, Stavros Stavrou

**Affiliations:** 1 Department of Electrical Engineering, Eindhoven University of Technology, 5600 Eindhoven, The Netherlands; 2 School of Computing, Edinburgh Napier University, Edinburgh EH10 5DT, UK; 3 Faculty of Pure and Applied Sciences, Open University of Cyprus, Nicosia 2220, Cyprus

**Keywords:** information fusion, polarization, multiple antenna, indoor positioning, localization, positioning accuracy, single access point positioning, fingerprinting

## Abstract

This research work investigates how RSS information fusion from a single, multi-antenna access point (AP) can be used to perform device localization in indoor RSS based localization systems. The proposed approach demonstrates that different RSS values can be obtained by carefully modifying each AP antenna orientation and polarization, allowing the generation of unique, low correlation fingerprints, for the area of interest. Each AP antenna can be used to generate a set of fingerprint radiomaps for different antenna orientations and/or polarization. The RSS fingerprints generated from all antennas of the single AP can be then combined to create a multi-layer fingerprint radiomap. In order to select the optimum fingerprint layers in the multilayer radiomap the proposed methodology evaluates the obtained localization accuracy, for each fingerprint radio map combination, for various well-known deterministic and probabilistic algorithms (Weighted k-Nearest-Neighbor—WKNN and Minimum Mean Square Error—MMSE). The optimum candidate multi-layer radiomap is then examined by calculating the correlation level of each fingerprint pair by using the “Tolerance Based—Normal Probability Distribution (TBNPD)” algorithm. Both steps take place during the offline phase, and it is demonstrated that this approach results in selecting the optimum multi-layer fingerprint radiomap combination. The proposed approach can be used to provide localisation services in areas served only by a single AP.

## 1. Introduction

During the last decade, several localization methods have been proposed [1,2,3], and with the advancement of computational power and the introduction of new technologies, more sophisticated localization solutions have emerged. These solutions typically include hybrid approaches where information from different sources are fused, aiming to improve localization accuracy. Such approaches may combine different wireless technologies, such as Wi-Fi and Bluetooth Low Energy (BLE) beacons [4,5], Wi-Fi with Visual Light Positioning (VLP) [6], or even VLP with ultrasound [7]. Localization approaches may also fuse data retrieved from maps [8], inertial, magnetic, or other sensors’ input [9,10,11]. In all cases, and depending on the technology and equipment available, the localization methodology utilizes parameters such as Received Signal Strength (RSS), Time of Flight (TOF), and Angle of Arrival (AOA). Methods based on TOF and AOA require complex equipment able to measure time (TOF) or signal arrival angles (AOA) with high accuracy. These processes may also require synchronization of transceivers and calibration of antennas [12].

When it comes to RSS localization, the popularity of the method lies on the utilization of existing wireless communication infrastructure, the simplicity of the localization algorithms, and the achieved levels of accuracy. In RSS fingerprint-based localization, the procedure involves an offline and an on-ine phase. During the offline phase, a dataset of RSS fingerprints, named *a radiomap*, is generated by recording the RSS value of each Access Point (AP) at multiple locations in the area of interest. The RSS values are then calibrated in order to improve the quality of the radio-map [13]. It has been shown that such datasets can be generated rapidly and at a relatively low cost, through the use of a deterministic radio propagation simulator [8,14,15]. The conventional approach involves performing time-consuming measurement campaigns in the actual environment [14]. The generated radio-map from such a campaign represents a static snapshot of the APs RSS values at each measured location. It has to be noted that every time the environment or the wireless network is modified, one requires a repeat of the measurements. During the online phase, the Mobile Station (MS) performs real-time RSS measurements. Different positioning algorithms are then applied to identify the best match between the observed RSS fingerprint and the respective mean value of the fingerprints recorded in the radiomap during the offline phase. An overview of typical fingerprint-based methods is provided in [16].

A significant drawback of these systems is that they require the deployment of several APs in order to generate unique fingerprints at each location, in order to achieve satisfactory positioning accuracy [15]. Due to this characteristic, RSS fingerprint-based localization systems cannot be easily utilized in small-scale environments, that is, residential places or open plan spaces, where a single AP is deployed.

In this paper, we examine the generation of unique fingerprint radiomaps from a single, multi-antenna AP by exploiting antenna polarization effects and combining these radiomaps into a multi-layer radiomap concept. Result analysis indicates that each antenna from the AP device can be considered as an independent transmitting source. Different polarization configurations are then utilized in order to ensure that RSS values from different antennas, at the same location, are not correlated. The proposed method’s performance is evaluated in a two-step procedure, initially by implementing and testing the performance of deterministic and probabilistic algorithms (Weighted k-Nearest-Neighbor—WKNN and Minimum Mean Square Error—MMSE) for a set of different antenna orientations, hence different polarization set-ups. Subsequently, the uniqueness of all candidate radiomaps is examined by calculating the correlation level of each fingerprint pair. High correlation scores indicate high probable localization errors, and vice versa. This method requires the implementation of the “Tolerance-Based Normal Probability Distribution (TBNPD)” algorithm [15].

The rest of the paper is organized as follows: Section 2 includes related research work on the impact of polarization. In the same section, an overview of RSS fingerprint-based localization methods and general evaluation techniques are included. In Section 3, the proposed approach is analyzed in more detail. Section 4 provides a description of the testing environment and the simulation parameters. Section 5 focuses on result analysis and performance evaluation. Finally, Section 6 draws conclusions and provides suggestions for future work.

## 2. Related Work

This section provides an overview of fingerprint-based localization methods and the related work that considers multipath and polarization effects in indoor localization procedures. The section also summarizes relevant localization evaluation techniques.

### 2.1. Fingerprint-Based Positioning Methodologies

Since the proposed approach primarily focuses on indoor RSS-based positioning, the main localization methods corresponding to such types of systems are presented below.

Two main types of localization algorithms are used in RSS-based indoor positioning platforms—deterministic and probabilistic. The *deterministic* algorithms estimate location $\hat{\ell }$ as a convex combination of the *K* reference locations [17]. $\hat{\ell }$ is calculated as the shortest distance between ${\bar{r}}_{i}$ and *s* in the *n*-dimensional space by using the following equation:

(1) $$\hat{\ell }=\sum_{i=1}^{K}\left(\frac{w_{i}}{\sum_{j=1}^{K}w_{j}}\ell_{i}^{\prime }\right)$$

The data set $\left\{\ell_{1}^{\prime },\ldots ,\ell_{l}^{\prime }\right\}$ denotes the reference locations between the respective fingerprint ${\bar{r}}_{i}$ and the observed measurement during positioning *s*, with respect to an increasing distance—that is, $∥{\bar{r}}_{i}-s∥$. The distance can be calculated using standard norms, such as the Manhattan (1-norm) [18], the Euclidean (2-norm) [19], or the Mahalanobis norm [20].

In its simplest form, the prescribed algorithm can assume K=1, resulting in the simple Nearest Neighbour (NN) method [19,21]. If several candidate locations are taken into consideration, but with equal weight factor $w_{i}$, the formula represents the *K*-Nearest Neighbour (KNN) method. In more complex environments, for improved accuracy, the full implementation of the Weighted *K*-Nearest Neighbour (WKNN) [18] is employed by setting the non-negative weight factor $w_{i}$ in Equation (1) as the inverse of $∥{\bar{r}}_{i}-s∥$.

In probabilistic methods, location *ℓ* is estimated by calculating and maximising the conditional posterior probabilities $p(\ell_{i}|s),\;i=1,\ldots ,l$ given an observed fingerprint *s* and a fingerprint database. In order to estimate the expected value of *ℓ*, the method may implement either the Maximum A Posteriori (MAP) [22] or the Minimum Mean Square Error (MMSE) approach [23].

The posterior probability $p(\ell_{i}|s)$ is obtained by applying Bayes’ rule:

(2) $$p\left(\ell_{i}|s\right)=\frac{p\left(s|\ell_{i}\right)p\left(\ell_{i}\right)}{\sum_{i=1}^{l}p\left(s|\ell_{i}\right)p\left(\ell_{i}\right)},$$

where $p(s|\ell_{i})$ is a conditional probability calculated through statistics at the survey stage, and $p(\ell_{i})$ is the a priori probability. The a priori probability is a weighting factor based on the probability distribution of the target over the reference position candidates that exist in the fingerprint data set. Similarly to the deterministic method, the formula can be simplified if no prior knowledge is assumed. In such a case, this *prior* can be assumed to be a unity, meaning that an equal a priori probability exists for all fingerprint candidates.

Both deterministic and probabilistic methods are implemented in more sophisticated fingerprint-based hybrid solutions, where data and information are fused in an effort to improve localization accuracy. Such approaches may combine different wireless technologies, such as Wi-Fi and Bluetooth Low Energy (BLE) beacons [4,5], Wi-Fi with Visual Light Positioning (VLP) [6], and VLP with ultra sound [7]. Hybrid solutions are also presented, including utilization of map information [8], inertial data, or other sensors’ input [9].

### 2.2. Mitigating Multipath and Polarization Effects

Research on polarization and multipaths referring to indoor localization techniques tends to focus on the minimization of their influence on the received signal, instead on how to use polarization to improve the localization process. In this scope, authors of [24] presented a method for decreasing errors of TOA-based indoor positioning systems, based on directional antennas with small side lobes.

Authors of [25] investigated the effects of polarization on the accuracy of an indoor location tracking system, and established an experimental model that includes parameters which take into account environmental effects. Based on their observations, they concluded that the accuracy of the location estimation is mainly dependent on the accuracy of the range measurements and the antenna polarization angle, which influence RSS, and thus, range accuracy.

In [26], another approach is presented where researchers investigated potential accuracy improvements in the RSS indoor localization process, through the introduction of directional antennas in the radio network infrastructure. The position and orientation of the directional antenna was carefully selected in order to decrease the correlation levels of the RSS fingerprints that form the *radiomap*.

The research community also proposed sophisticated and specialized RF designs for enabling spatial re-usability, as well as polarization diversity to mitigate multipath propagation. Authors of [27,28] designed a switched beam array optimized for 2.45 GHz wireless indoor applications. The proposed antenna appears to support 2D target localization using measurements from a single anchor node, achieving an average localization error of 1.7 m.

Polarization Scenarios were also investigated through ultra wide-band (UWB) fingerprinting [29]. Comparisons between vertical and horizontal polarization cases at a frequency range from 3 GHz to 11 GHz suggested that horizontal polarization provides greater accuracy than vertical polarization.

The usage of linear and circular polarized antennas for indoor RSS positioning techniques was proposed in [30]. In this work, it was shown that the utilization of linear and circular polarized antennas, instead of only linear polarized antennas, decreases the standard deviation of the received power and enhances the effective range.

All the aforementioned work focuses on mitigating multipath and polarization effects, rather than utilizing their inherent properties for the benefit of improving RSS localization accuracy. To the authors’ best knowledge, no previous research has investigated how polarization can be utilized to provide low correlation fingerprints.

### 2.3. RTLS Performance Evaluation Techniques

A typical RTLS evaluation method refers to the development of benchmark standards for the comparison of the performance of different localization schemes [31]. Such schemes usually include environment type categorization, as well as their dynamic behaviour. The main disadvantages of these benchmarks are the complexity and abstract procedures that need to be implemented.

More precise methods were presented by researchers in [32,33], who suggested the enumeration of a number of critical factors that influence the performance of localization platforms. These factors include the number of transmitters, the number of reference measurements, and the signal measurement dynamics. They examined a set of localization mechanisms and evaluated their performance robustness under various configuration settings using two typical types of building environments: an office building and an underground floor-plan. Although they enumerated several critical factors, they did not provide a direct relation between these factors. The difficulty of assessing the performance of different localization systems was also reported in [34].

The performance of radiomaps was also exploited in [35]. In this research, radiomaps were generated from dynamically collected measurements during the offline phase. The KNN algorithm was then employed for positioning during the online phase. In the dynamic data collection process, signals were collected automatically, while collectors were moving along the pre-designed paths. During the conventional data collection process, the measurements were collected statically: first, the service area was divided into pre-designed cells, which usually had a rectangular shape; then, the collectors gathered signals for each cell, until a sufficient number of samples were collected. Although more accurate, this is a much more laborious and cumbersome data collection process. The positioning performance in the case of the dynamic collection process was compared with different grid spacing, and with various K numbers for a KNN algorithm. However, the proposed methodology is algorithm-specific, and the general outcome was that the positioning performance is affected by various parameters that should be thoroughly decided. Authors do not define such critical factors, and they also do not propose a holistic evaluation methodology.

In order to assess any positioning platforms which have already been deployed, in a previous study we proposed the application of binomial distribution [36]. To evaluate of the quality of RSS fingerprint databases, we also developed a dedicated correlation algorithm, named the “Tolerance-Based Normal Probability Distribution (TBNPD)” [15]. This algorithm calculates the correlation level for every pair of fingerprint entries forming the radiomap, while also considering possible RSS fluctuations ($RSS_{tol}$), occurring due to the dynamic nature of the environment. The TBNPD algorithm offers the possibility to assess the uniqueness of each fingerprint entry in a radiomap, prior to its utilization in a Real-Time Locating System (RTLS). For this reason, the TBNPD algorithm is considered suitable and convenient to be used for the assessment of the candidate radiomaps generated in the current paper.

Based on the analysis of this algorithm, the Correlation Score ($CS_{pairAB}$) of any pair of random fingerprint entries, A $\left(x_{A},y_{A}\right)$ and B $\left(x_{B},y_{B}\right)$, for any active AP ($AP_{i}$), without introducing the $RSS_{tol}$ parameter, is given by the formula:

(3) $$CS_{pairAB}=\frac{1}{\sigma_{{\bar{RSS}}_{AP_{i}}}\sqrt{2\pi }}e^{-\frac{1}{2}{\left(\frac{{\bar{RSS}}_{B}-{\bar{RSS}}_{A}}{\sigma_{{\bar{RSS}}_{AP_{i}}}}\right)}^{2}}$$

where random fingerprint entries $A,B\in Area_{radiomap}$, and $\sigma_{{\bar{RSS}}_{AP_{i}}}$ is the standard deviation of the $\bar{RSS}$ values observed from each $AP_{i}$ as:

(4) $$\sigma_{{\bar{RSS}}_{AP_{i}}}=\sqrt{\frac{\sum_{i=1}^{n}{\bar{RSS}}_{i}^{2}-{\left(\frac{\sum_{i=1}^{n}{\bar{RSS}}_{i}}{n}\right)}^{2}}{n-1}},$$

where *n* is the number of fingerprint entries in the radio map and ${\bar{RSS}}_{i}\geq MS_{Sensitivity}$.

By introducing the $RSS_{tol}$ parameter in Formula (3), the correlation score $CS_{TBNPD}$, is formulated. The $CS_{TBNPD}$ for a pair of random points $A,B\in Area_{radiomap}$ and for any $AP_{i}$ is calculated by (5) below:

(5) $$CS_{TBNPD_{pairAB}}=\frac{1}{\sigma_{{\bar{RSS}}_{AP_{i}}}\sqrt{2\pi }}e^{-\frac{1}{2}{\left(\frac{{\bar{RSS}}_{diff}}{\sigma_{{\bar{RSS}}_{AP_{i}}}}\right)}^{2}}$$

where

(6) $${\bar{RSS}}_{diff}=\left|{\left({\bar{RSS}}_{A}\right)}_{AP_{i}}-{\left({\bar{RSS}}_{B}\right)}_{AP_{i}}\right|-2RSS_{tol}.$$

In Formula (6), ${RSS}_{diff}\geq 0$. For ${RSS}_{diff}<0$, the value was set to 0, since the range of the RSS values of the two fingerprint entries overlap, indicating a high level of correlation.

The total correlation score ($CS_{TBNPD_{total}}$) to be utilized to assess any candidate radiomap is the product of the correlation scores of all active APs:

(7) $$CS_{TBNPD_{total}}=\prod_{AP=1}^{m}\left(CS_{TBNPD_{pairAB}}\right).$$

## 3. Proposed Approach

### High-Level Description of the Proposed Approach

In this paper, we present a new localization approach based on a single, multi-antenna AP, where each AP antenna acts as a separate transmitter. By carefully configuring the orientation and polarization of each antenna, one can influence the RSS values that form the fingerprint radiomap. RSS from different antennas can then be combined in order to generate a *multi-layer radiomap*. The multi-layer radiomap is defined as the unified data set created, from the fusion of RSS and antenna identification data recorded at each receiver location. In order to practically achieve such a task, the APs’ wireless drivers should be capable of supporting *Radiotap*, which is a de facto standard for 802.11 frame injection and reception. *Radiotap* offers the capability to retrieve additional information about 802.11 frames, from the driver to userspace applications, and defines two important antenna fields: Firstly, the *IEEE80211_RADIOTAP_ANTENNA* field, a unit-less indication of the Rx/Tx physical antenna identification (Antenna ID), which is a parameter important for identifying the exact transmitting source of the RSS recorded by the Mobile User (MS); and secondly, the *IEEE80211_RADIOTAP_DBM_TX_POWER* field, which provides the RF signal power. A typical code snippet of transmission definitions for the Atheros driver is presented below:

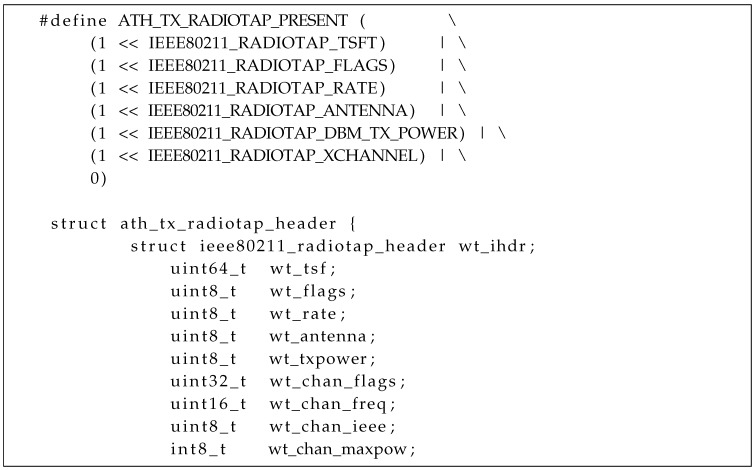



**Figure 1:**
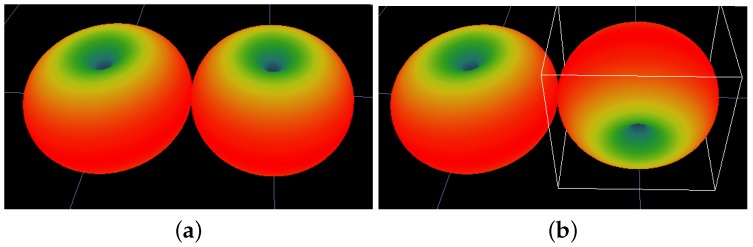
Typical antenna orientation set-ups: Part 1. (**a**) Tx-01 Vertical -Tx-02 Vertical; (**b**) Tx-01 Vertical -Tx-02 Horizontal 90deg.

**Figure 2:**
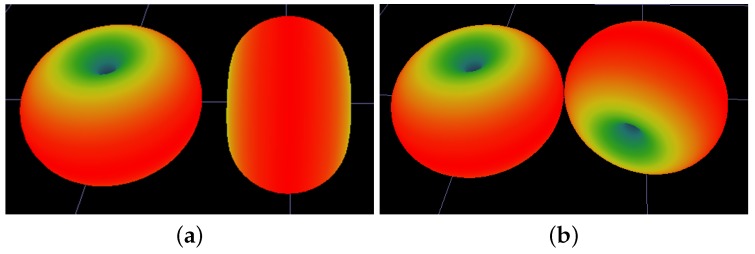
Typical antenna orientation set-ups: Part 2. (**a**) Tx-01 Vertical -Horizontal 0deg; (**b**) Tx-01 Vertical -Horizontal 45deg.

As mentioned earlier, each combination of different antenna orientations will potentially generate a different candidate radiomap. For example, in the case of a single AP with two dipole antennas, a Scenario could include both antennas to be set in the vertical-z axis direction. Another Scenario may assume that the first antenna is kept at the vertical orientation, while the second antenna is at a horizontal orientation pointing at zero degrees. A different Scenario may maintain the first antenna at the vertical orientation, and the second antenna to be reconfigured at 45 degrees or 90 degrees at the horizontal level, and so forth. Scenarios may also assume different antenna orientations at the receiver site. The aforementioned typical Scenarios are illustrated in Figure 1 and Figure 2.

After preparing the desired Scenarios, simulations were run in order to generate the respective radiomaps. Each radiomap was evaluated through various localization algorithms, noting the resulting positioning error. Based on the results, the best performing radiomap was finally selected to be implemented in the RSS-based fingerprint localization platform. For the purposes of our experiment, we utilized TruNET wireless, a deterministic radio planning simulator.

Initially, the area of interest was designed in a *TruNET wireless* simulator, taking into consideration the different building structure geometries, dimensions, and material constitutive parameters. A list of different Scenarios was also created by configuring the wireless network antennas, as described previously. The wireless network consisted of a single Access Point (AP) with a minimum of two monopole antennas. The proposed approach is applicable to any antenna type.

Afterwards, a simulation took place for each Scenario, and a candidate multi-layer radiomap was generated based on the fused antenna information. More specifically, each AP antenna was considered as a separate transmitter. The calculated RSS values, linked with the transmitting source (Antenna ID), were registered in the receiver cells. Depending on the Scenario set-up (different combination of antenna orientations/polarization), the RSS values were expected to vary significantly. The candidate radiomaps were then exported to a standardized template format, and their localization accuracy assessed.

In order to perform the assessment, a testing dataset sample was required, which is a set of RSS fingerprints at a number of a priori known locations within the study area. Such a sample could either be retrieved during the online phase, as described in Section 2.1, or can be generated through a simulation. The size of the aforementioned sample and the distribution of the test locations were both selected in such a way as to ensure reliable and objective testing, as per [13].

For the purpose of the radiomap assessment, two types of localization algorithms were implemented: a deterministic (WKNN) and a probabilistic (MMSE) algorithm. Each candidate radiomap was tested and its performance evaluated with respect to the achieved localization accuracy. Lastly, the correlations between all pairs of fingerprints in the dataset were examined, and an overall correlation score for the candidate radiomaps was calculated [15]. A high correlation score designates a higher probable localization error, due to high similarities between different fingerprint pairs (locations). After the completion of the aforementioned iterative procedure, the most suitable radiomap among the candidates was selected. The selection was based on two performance metrics: ‘minimum mean localization error’ and ‘minimum correlation score’.

## 4. Test Environment

This section analyses realistic multipath Scenarios. The Scenarios refer to a laboratory area of approximately 100 m${}^{2}$, which approximates a typical residential indoor floor. The 3D environment and the various different wireless network set-ups were simulated in *TruNET wireless*, a 3D polarimetric ray-tracing simulator [37]. The transmitter was a single AP with two dipole antennas, transmitting 20 dBm at 2.4 GHz, which is a typical IEEE 802.11 setup. The aforementioned transmitter was placed in the room most north-east of the laboratory, at a height of 2.2 m, in order to minimize the LOS area as much as possible and allow the multipath and fast fading effects to have a higher impact. The building structure and large furniture were configured using material constitutive parameters obtained from the literature [38], as per Table 1.

The generated candidate radiomaps include 406 receiver cells at a height of 1.2 m. A series of 11 different Scenarios (numbers 2 to 12) were created by combining different polarization set-ups. The 11 Scenario configuration parameters are shown in Table 2.

Finally, for reference purposes, a typical Scenario (No. 1) with five APs was created for the same environment. Results were compared with the 11 single AP Scenarios.

**Figure 3:**
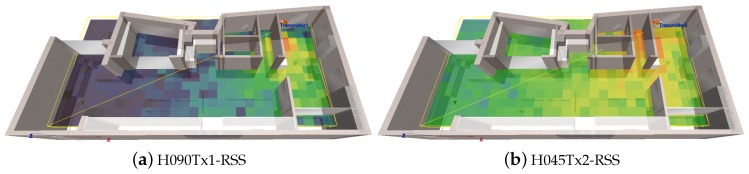
Radiomap vs. antenna polarization, H090 and H045.

**Figure 4:**
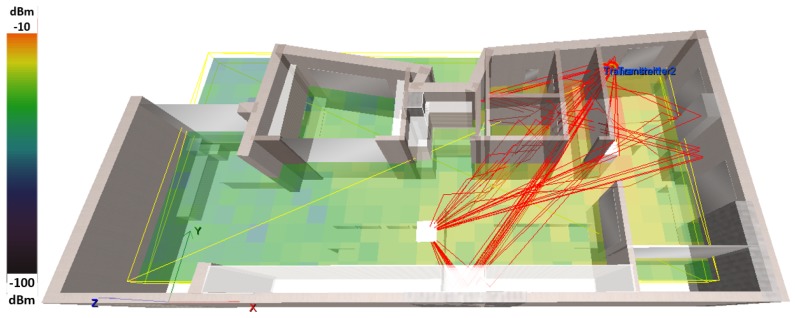
Multipath effect in indoor environment (Rx Cell 198).

**Figure 5:**
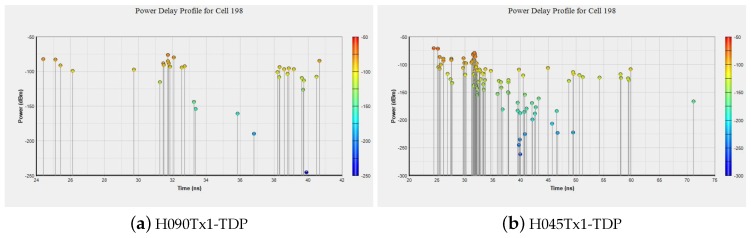
Time delay profile vs. antenna polarization, H090 and H045.

The effects of polarization, antenna orientation and multipath are depicted in the snapshots of Figure 3 and Figure 4. In Figure 5, we present the changes in Power Delay Profile (PDP) for a randomly selected cell. It can be easily observed that the PDP may vary due to the changes in polarization and antenna orientation.

## 5. Performance Evaluation

**Figure 6:**
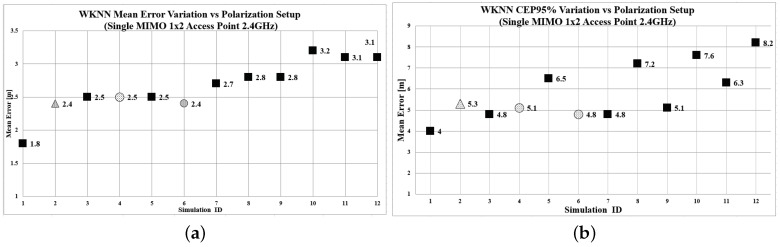
WKNN Algorithm performance. (**a**) Mean error per polarization Scenario; (**b**) CEP95 per polarization Scenario.

**Figure 7:**
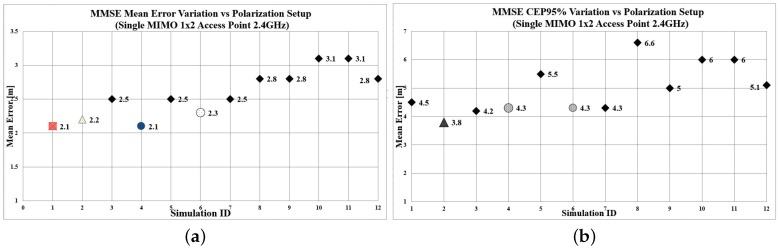
MMSE algorithm performance. (**a**) Mean error per polarization Scenario; (**b**) CEP95 per polarization Scenario.

**Figure 8:**
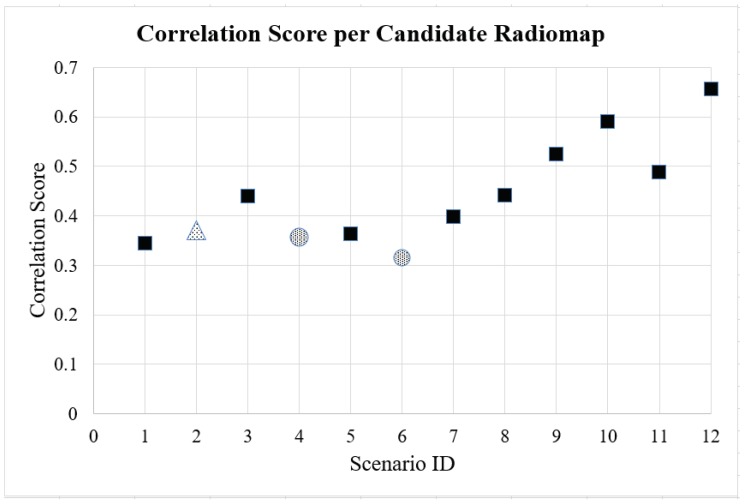
Correlation score per Scenario.

The proposed methodology was implemented for all 11 Scenarios. For testing purposes, the sample size of the test points was determined at 48 for WKNN and 53 for MMSE localization algorithms. The aforementioned samples were selected with a “Simple Random Sample” procedure, as presented in [13]. The calculated mean error and Circular Error Probable (CEP) 95% for both WKNN and MMSE localization algorithms are presented in Table 3 and Figure 6 and Figure 7, respectively. The quality correlation score (CS) for each radiomap is illustrated in Figure 8.

Based on the aforementioned results, a significant variation of the localization accuracy can be observed, which depends on the transmitter configuration setup. Scenarios of special interest are Numbers 4 and 6 (marked with a circle) and Scenario No. 2, illustrated with a triangular marker. When implementing WKNN (*k* = 4), the mean error varies from 1.69 m for Scenario No. 6, and 1.91 m for Scenarios 2 and 4, to 3.25 m for Scenario No. 12. Similar behaviour can be noted for the mean error achieved when utilizing the MMSE (σ = 9) algorithm. We recorded a minimum mean error of 2.11 m for Scenario No. 4 and 2.29 m for Scenario No. 6, to a maximum of 3.09 m for Scenario No. 11. The quality evaluation of the candidate radiomaps supports the above findings, indicating that the most appropriate radiomaps in this setup are No. 6 (CS = 0.314) and No. 4 (CS = 0.355). The localization accuracy that can be achieved with the proposed methodology is comparable with the benchmark Scenario No. 1, where five APs were used. More specifically, although utilizing the information from five different APs leads to a minimum of 1.04 m mean error, the 1.69 m error still able to be accomplished by a single, two-antenna AP appears to be satisfactory for performing RSS fingerprint localization in small areas, like residential environments. Typical residential applications may include remote monitoring of patients, elderly people, children, and animals. Such localization solutions could be also integrated in smart homes, where different sensors can be activated depending on the proximity of the user. Another observation that is worth mentioning is related to Scenario 2, where the configuration of both antennas was set to Vertical Polarization. The RSS differences occurred only due to the spacial separation of the antennas. On the other hand, it is noted that Scenario numbers 4 and 6, which performed best, were configured with antennas at 45 degrees. Finally, it can be safely assumed that by utilizing various MIMO configurations and a proper antenna polarization configuration, localization accuracy will be improved.

## 6. Conclusions

In this paper, a novel indoor RSS localization approach was presented. The novelty of the approach lies on the generated RSS, resulting from the various antenna polarisation states, and the antenna elements direction of a single AP system. Testing and evaluation of the proposed methodology indicates that high-quality radiomaps can be generated, leading to satisfactory localization accuracies. The work demonstrates the ability to implement RSS-based Real-Time Localisation Systems (RTLS) in areas where only a single multi-antenna AP exists. This research may be further expanded by investigating various MIMO configurations.

## Figures and Tables

**Table 1:** Material constitutive parameters of the test environment.

Material	El.Per. (F/m)	L. Tangent
**Concrete**	3.9	0.23
**Wood**	2	0.025
**Brick**	5.5	0.03
**Metal**	1	1,000,000
**Plasterboard**	3	0.067
**Glass**	4.5	0.007

**Table 2:** Antenna configuration per Scenario ID.

Scenario ID	2	3	4	5	6	7	8	9	10	11	12
Antenna 1 Pol	V	H$0^{\circ }$	H$45^{\circ }$	H$90^{\circ }$	H$135^{\circ }$	H$180^{\circ }$	H$0^{\circ }$	H$0^{\circ }$	H$0^{\circ }$	H$0^{\circ }$	H$0^{\circ }$
Antenna 2 Pol	V	V	V	V	V	V	H$0^{\circ }$	H$45^{\circ }$	H$90^{\circ }$	H$135^{\circ }$	H$180^{\circ }$

**Table 3:** Localization algorithm performance and correlation score per candidate radiomap.

Decision Factor	2	3	4	5	6	7	8	9	10	11	12
WKNN Mean Error (m)	1.91	2.21	1.91	1.98	1.69	2.09	2.27	2.72	3.01	2.67	3.25
WKNN CEP 95% (m)	3.41	4.27	3.69	3.36	3.45	3.48	6.22	4.45	2.99	9.00	9.85
MMSE Mean Error (m)	2.17	2.48	2.11	2.49	2.29	2.55	2.82	2.71	3.08	3.09	2.52
MMSE CEP 95% (m)	3.78	4.14	4.19	5.60	4.27	4.37	4.97	4.54	6.07	5.86	5.01
Correlation Score	0.371	0.440	0.355	0.363	0.314	0.398	0.442	0.525	0.591	0.488	0.657

## Abbreviations

The following abbreviations are used in this manuscript:

AOA	Angle of Arrival
AP	Access Point
BLE	Bluetooth Low Energy
IoT	Internet of Things
KNN	K Nearest Neighbour
MAP	Maximum A Posteriori
MDPI	Multidisciplinary Digital Publishing Institute
MMSE	Minimum Mean Square Error
MS	Mobile Station
NN	Nearest Neighbour
RSS	Received Signal Strength
RTLS	Real Time Localization System
TDOA	Time Difference of Arrival
TOA	Time of Arrival
TOF	Time of Flight
VLP	Visual Light Positioning
WKNN	Weighted K Nearest Neighbour
